# Effect of Pyroptosis-Related Genes on the Prognosis of Breast Cancer

**DOI:** 10.3389/fonc.2022.948169

**Published:** 2022-07-25

**Authors:** Ying Zhou, Jianfeng Zheng, Mengru Bai, Yuzhen Gao, Nengming Lin

**Affiliations:** ^1^ Department of Clinical Pharmacology, Key Laboratory of Clinical Cancer Pharmacology and Toxicology Research of Zhejiang Province, Affiliated Hangzhou First People’s Hospital, Cancer Center, Zhejiang University School of Medicine, Hangzhou, China; ^2^ Department of Clinical Pharmacy, Affiliated Hangzhou First People’s Hospital, Zhejiang University School of Medicine, Hangzhou, China; ^3^ Department of Obstetrics and Gynecology, Affiliated Hangzhou Hospital, Nanjing Medical University, Hangzhou, China; ^4^ Department of Clinical Laboratory, Sir Run Run Shaw Hospital of Zhejiang University School of Medicine, Hangzhou, Zhejiang, China; ^5^ Translational Medicine Research Center, Affiliated Hangzhou First People’s Hospital, Zhejiang University School of Medicine, Hangzhou, China

**Keywords:** breast cancer, algorithm, programmed cell death, bioinformatic analyses, PRGs

## Abstract

**Backgrounds:**

Pyroptosis, a newly pattern of specific programmed cell death, has been reported to participate in several cancers. However, the value of pyroptosis in breast cancer (BRCA) is still not clear.

**Methods:**

Herein, we analyzed the data of BRCA from both The Cancer Genome Atlas (TCGA) and GSEA MSigDB database. Based on the obtained pyroptosis-related genes (PRGs), we searched the interactions by STRING. After that, we performed clustering analysis by ConsensusClusterPlus. The PRGs with significant prognostic value were then screened through univariate cox regression and further evaluate by constructing a risk model by least absolute shrinkage and selection operator (LASSO) Cox regression. The immune and sensitivity to drugs were also predicted by comprehensive algorithms. Finally, real-time quantitative PCR (qPCR) was performed on two of the screened signature PRGs.

**Results:**

A total of 49 PRGs were obtained from public database and 35 of them were significantly differentially expressed genes (DEGs). Cluster analysis was then performed to explore the relationship between DEGs with overall survival. After that, 6 optimal PRGs (GSDMC, IL-18, CHMP3, TP63, GZMB and CHMP6) were screened out to construct a prognostic signature, which divide BRCA patients into two risk groups. Risk scores were then confirmed to be independent prognostic factors in BRCA. Functional enrichment analyses showed that the signature were obviously associated with tumor-related and immune-associated pathways. 79 microenvironmental cells and 11 immune checkpoint genes were found disparate in two groups. Besides, tumor immune dysfunction and exclusion (TIDE) scores revealed that patients with higher risk scores are more sensitive to immune checkpoint blockade treatment. Patients in the low-risk group were more sensitive to Cytarabine, Docetaxel, Gefitinib, Paclitaxel, and Vinblastine. Inversely, patients in the high-risk group were more sensitive to Lapatinib. Finally, we found that, CHMP3 were down-regulated in both BRCA tissues and cell lines, while IL-18 were up-regulated.

**Conclusion:**

PRGs play important roles in BRCA. Our study fills the gaps of 6 selected PRGs in BRCA, which were worthy for the further study as predict potential biomarkers and therapeutic targets.

## Introduction

Breast cancer (BRCA) remains the most common and leading deadliest malignancy of women worldwide, despite huge advances in epidemiological, laboratory and clinical research (1). 70–80% of BRCA patients with early-stage and non-metastatic could be cured (2). Early diagnosis has been proved to effectively improve the cure rate of BRCA, about 30% of early-stage patients developed into advanced malignant BRCA with distant organ metastases. The advanced BRCA is considered incurable, in which the median overall survival time was only 2-3 years and the 5-years survival rate was only 20% (3, 4). Therefore, there is an urgent need to understand the molecular mechanism underlying of BRCA, and then identify and characterize specificity and sensitivity biomarkers for BRCA diagnosis, treatment and prognosis.

Pyroptosis was firstly found in 1986 (5), and defined as a new pattern of specifically programmed cell death in 2001 (6). It could be characterized by continuous cell swelling, bubble-like protrusions formation on the cell membrane surface before its rupture and release of cell contents which could trigger a strong inflammatory response (7). Similar to apoptosis, chromatin condensation and DNA damage also appear in the progress of pyroptosis (8, 9). As we all know, tumors are skilled at escaping the cell death pathways including apoptosis, autophagy, necrosis and pyroptosis (10, 11). All these death pathways are fully studied for their roles in anticancer defense mechanisms except for pyroptosis. Pyroptosis was reported to participate in various pathogenesis of nervous system diseases (12), autoimmune diseases (13), and cardiovascular diseases (14). In recent years, more and more researches have been performed to focus on the relationship between cancers and pyroptosis. Studies suggested that pyroptosis might be involved in the early diagnosis and treatment of cancers. In addition, it was also reported to relate to the progress of drug resistance (15). However, the role of pyroptosis in BRCA remains unclear.

In our study, we found six prognostic pyroptosis-relate genes (PRGs) from a public database for BRCA. The PRG-signature based on the six PRGs was developed. In addition, we also found differences in enrichment pathways, immune checkpoints, immune microenvironment, and sensitivity to several chemotherapeutic agents between risk groups.

## Materials and Methods

### Data Collection

We obtained processed RNA sequencing and clinicopathological data from TCGA including 1075 BRCA samples and 113 normal ones. GSE20685 (16) including 327 BRCA samples with clinical prognostic information, and GSE42568 (17) including 17 normal samples and 104 BRCA samples with clinical prognostic information were downloaded from GEO database (18). PRGs were screened from GSEA-MSigDB database (19). We used t-test of R3.6.1 (http://127.0.0.1:15190/library/stats/html/t.test.html) to analyze PRGs expression differences in tumor and normal samples (p < 0.05). The expression values are hierarchically clustered using pheatmap (https://cran.r-project.org/web/packages/pheatmap/index.html) (20).

### Interactive Network Construction

Based on the PRGs obtained, the interactions among proteins were searched by STRING 11.0 (http://string-db.org/) (21). The interaction scores higher than 0.4 were selected as the screening threshold to construct the interaction network, which was visualized by Cytoscape 3.6.1 (http://www.cytoscape.org/) (22). The pearson correlation coefficients (PCCs) among the targeted PRGs were also calculated by cor function of R3.6.1 (http://77.66.12.57/R-help/cor.test.html). |PCC|>0.3 and P<0.05 was selected as the screening threshold to construct the co-expression network, which was also visualized by Cytoscape 3.6.1.

### Subtype Analysis

Based on significantly differentially expressed PRGs (DEPGs) selected above, ConsensusClusterPlus1.54.0 (23)(http://www.bioconductor.org/packages/release/bioc/html/ConsensusClusterPlus.html) was then applied to analyze subtypes of BRCA. Survival and prognosis of different BRCA subtypes were then assessed (24). Then, based on the obtained cancer subtypes, the R3.6.1 language survival pack version 2.41-1 was applied to evaluate the correlation between prognosis and different subtypes. The clinical information of different subtypes was then statistically plotted, and chi-square test was used to compare the distribution among different subtypes.

### Signature Development Based on PRGs With Significant Prognosis

After screening the prognostic PRGs by Univariate Cox regression (P<0.05) (24), the LASSO Cox regression model (25) in the R penalty package (26) and 1000 cross-validation analysis were used to screen out the best combination of PRG markers. In addition, a risk model for BRCA patients was constructed based on the following formula:


$$\text{Risk score}\left(\text{RS}\right)=\sum {\text{β}}_{\text{PRG}}\times {\text{Exp}}_{\text{PRG}}$$

In the formula, β_PRG_ represented the LASSO for optimized PRGs and Exp_PRG_ means the expression level of homologous PRGs in the TCGA-BRCA dataset. Calculate the RS of each BRCA patient, and use the calculated median RS as the cutoff value, and further divide the BRCA patients into high-risk groups and low-risk groups. In the TCGA-BRCA training set and GSE42568, GSE20685 validation data sets, the same method was applied to build the risk model. Subsequently, the Kaplan-Meier analysis (24) was used to evaluate the prognostic value between the two risk subgroups.

### Immunity Analysis and Sensitivity of Chemotherapy Drugs

The immune microenvironment is also closely related to the occurrence and development of BRCA. Based on single-sample gene set enrichment analysis (ssGSEA) (27), the enrichment fraction of 28 immune cells (28) was calculated to represent the relative abundance of each TME-infiltrated cell in BRCA samples using GSVA (29). In addition, three arithmetics, CIBERSORT (30, 31), xCELL (32), MCPcounter (33), were wielded to compare the difference in the proportion of various immune cells in different risk groups. Further, according to the expression data of the BRCA samples, the immune and stromal scores were estimated from R3.6.1 through ESTIMATE to represent the presence of matrix and immune cells (34). The expression levels of 13 immune checkpoint genes (BTLA, TNFRSF9, ICOS, PDCD1, TIGIT, CTLA4, LAG3, CD274, TNFRSF4, HAVCR2, SIRPA, CD47, and VTCN1) were extracted, and their expression differences in the risk group were compared by the intergroup t-test.

The potential response to immune checkpoint blockade (ICB) was predicted using the TIDE algorithm (35). We extracted chemotherapy drugs from the Genomics of Drug Sensitivity in Cancer database (https://www.cancerrxgene.org/) (36) and used R3.6.1 pRRophetic (37) to assess IC50 levels.

### HPA Analysis

Expression of PRGs showed by immunohistochemical (IHC) staining in both normal and BRCA tissuses were searched by the resource of the HPA database (https://www.proteinatlas.org/). For IL-18, normal tissue, NOS(M-00100), patient id (2773); breast lobular carcinoma (M-85203), patient id (2199). For CHMP3, normal tissue, NOS(M-00100), patient id (3856); Breast lobular carcinoma (M-85203), patient id (4229).

### Real-Time qPCR Analysis

MDA-MB-123 and MDA-MB-453 cells were purchased from the National Collection of Authenticated Cell Cultures (Shanghai, China) and incubated in the L-15 culture medium (Gibco, 41300039) with 10% fetal bovine serum. MCF-7 cells was culture in the MEM culture medium (Gibco, 41500034) with 1.5 g/L NaHCO3, 0.11 g/L sodium pyruvate (Invitrogen, 11360070) and 0.01 mg/mL bovine insulin (Sigma, 91077c-1G). MCF-10A purchased from American Type Culture Collection (ATCC, CRL-10317) was cultured in special culture medium (Procell, China) containing DMEM/F12, 5% HS, 20ng/mL EGF, 0.5μg/mL Hydrocortisone, 10μg/mL Insulin, 1% NEAA and 1% P/S. Real-time qPCR was performed by ABI7500 (Thermo Fisher, Singapore) after RNA extraction and reversed transcription from all these four cell lines. The primers we used for qPCR were listed in the **Additional file 1 Table S1**.

### Statistical Analysis

We used R package (v4.0.2), TBtools and GraphPad Prism (v8.0) to perform and visualize statistical analysis. Kaplan-Meier survival analysis was performed on the risk group by log-rank test to draw a survival curve. Wilcoxon test is used to compare the difference between two groups.

## Results

**Figure 1:**
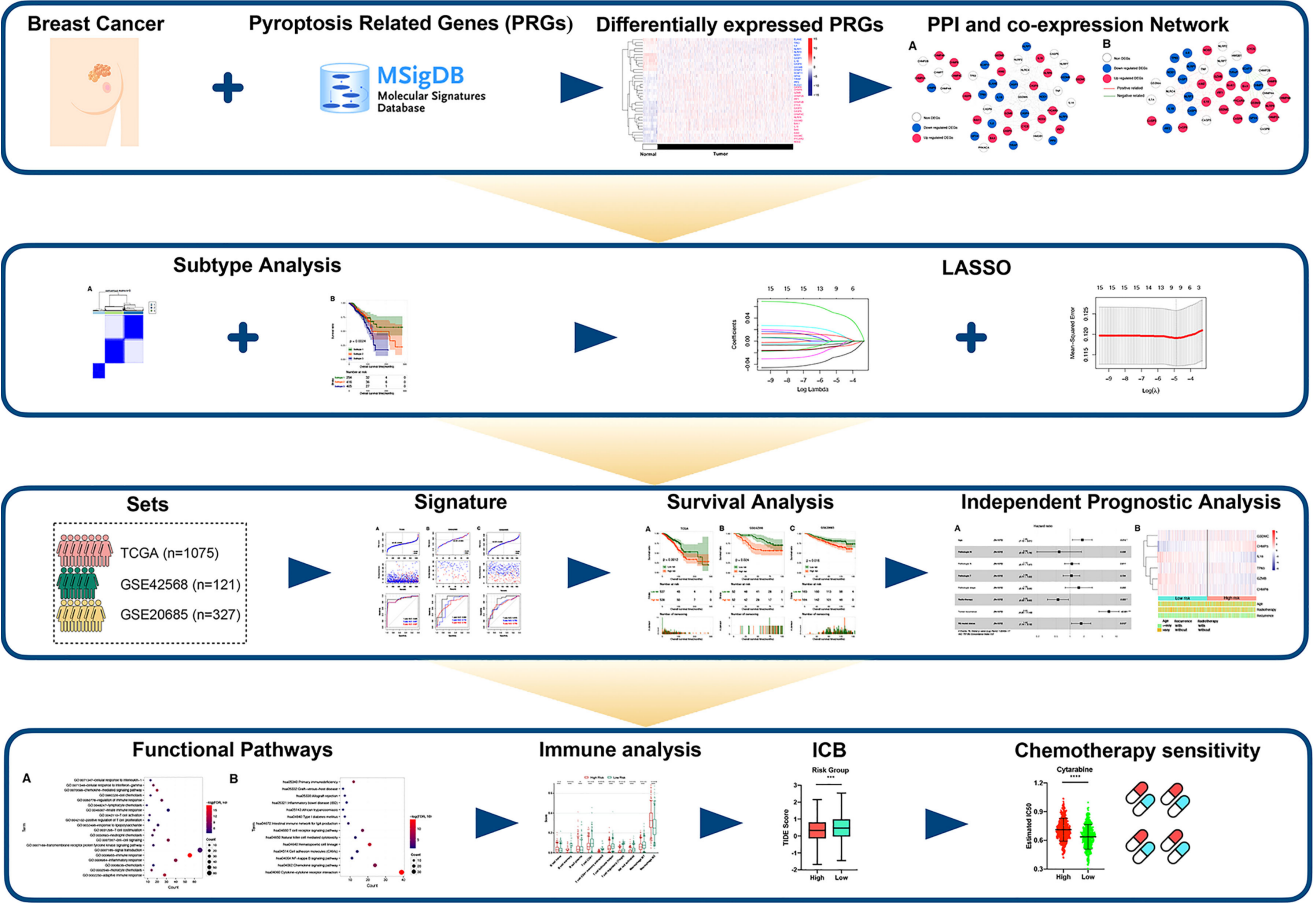
Flow diagram of our study.

**Figure 1** showed the flowchart we created for the entire study.

### Differentially Expressed PRGs (DEPGs)

**Figure 2:**
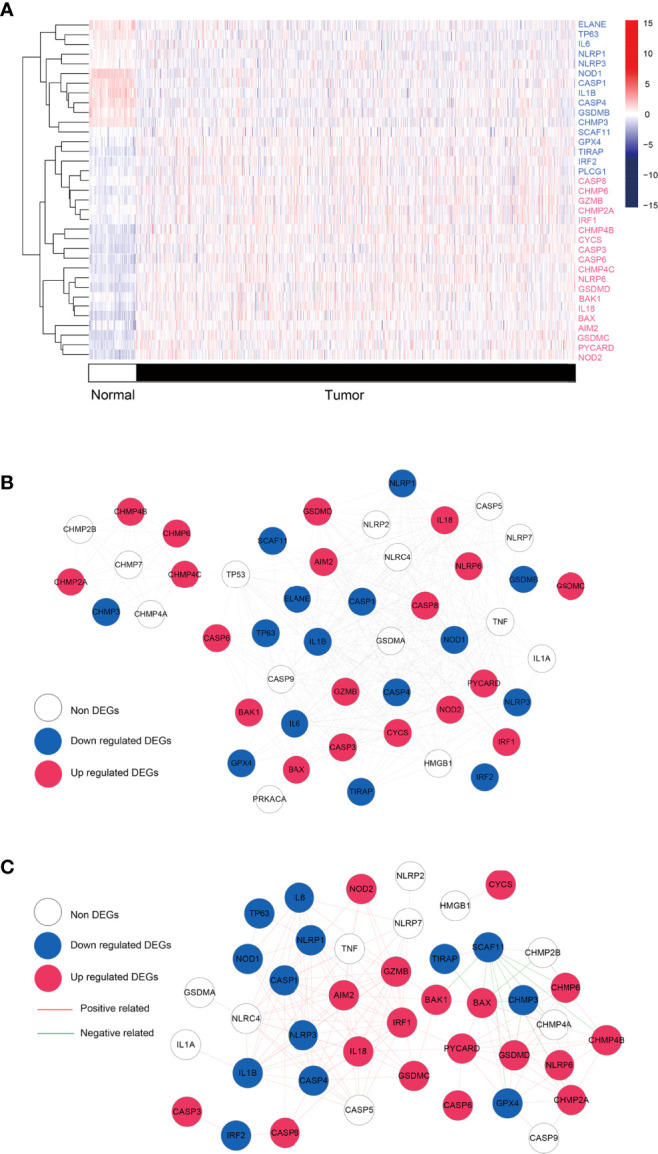
Differential analysis and network. **(A)** Heatmap showing the expression levels of pyroptosis-related genes with significant expression levels in breast cancer tumors and control samples. Red: up regulated; blue: down regulated. **(B)** Protei-protein interaction. Blue, red, and white nodes represent genes that are significantly down-regulated, up-regulated, and insignificantly differentially expressed in tumor tissue, respectively. **(C)** co-expression network based on pyroptosis-related genes. Blue, red, and white nodes represent significantly down-regulated, up-regulated, and insignificantly differentially expressed genes in tumor tissues, respectively. Red and green junctions represent significantly positive and negative correlations.

Combining the GSEA MSigDB and the attached documents of literature, we obtained a total of 49 PRGs. By comparing the expression differences of the above genes in BRCA and normal tissues, a total of 35 significantly DEPGs were screened. **Figure 2A** showed the clustering heat map of significantly DEPGs. Among them, 16 PRGs (ELANE, TP63, IL6, NLRP1, NLRP3, NOD1, CASP1, IL1B, CASP4, GSDMB, CHMP3, SCAF11, GPX4, TIRAP, IRF2, and PLCG1) were down-expressed in the tumor samples while 19 PRGs (CASP8, CHMP6, GZMB, CHMP2A, IRF1, CHMP4B, CYCS, CASP3, CASP6, CHMP4C, NLRP6, GSDMD, BAK1, IL-18, BAX, AIM2, GSDMC, PYCARD, and NOD2.) were over-expressed in the tumor samples. Then, we conducted a protein-protein interactions (PPIs) analysis by using the STRING database to explore further the interactions of these PRGs, 310 pairs of PPIs were obtained to construct the interaction network (**Figure 2B** and **Additional file 2 Table S2**). A total of 300 co-expression connection pairs were obtained to construct the co-expression network (**Figure 2C** and **Additional file 3 Table S3**).

### Subtype Analysis

**Figure 3:**
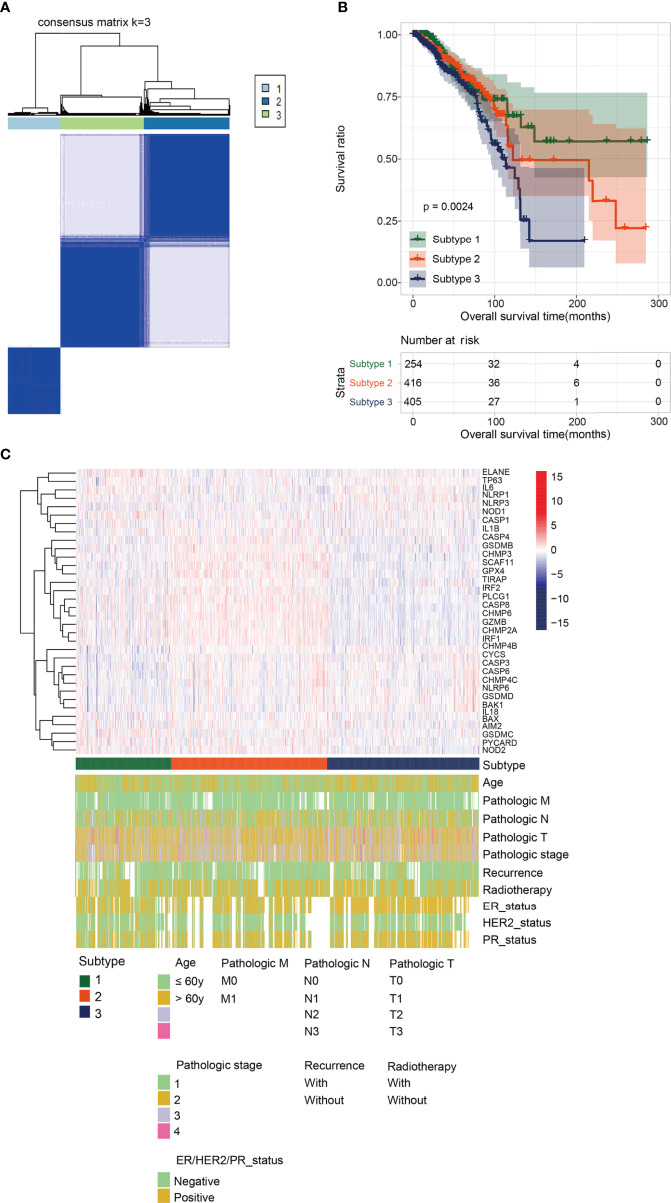
Subtype analysis. **(A)** Cluster diagram for subtype analysis of breast cancer samples. The intragroup correlations were the highest and the inter-group correlations were low when k=2. **(B)** The Kaplan-Meier analysis for the three different subtypes. Subtype 1 had the best prognosis, while Subtype 3 had the worst prognosis. **(C)** The distribution map of pyroptosis- related genes and clinicopathologic characters in the three subtypes.

Based on 35 significantly DEPGs screened out above, subtype analysis of BRCA was performed. When k=3 (k: clustering variable), the correlation within the group was the highest, and the correlation between the groups was low, a total of three different subtypes were obtained therewith (**Figure 3A**). Subtype1, 2, and 3 contained 254, 416, and 405 tumor samples, respectively. We then assessed the survival outcomes of patients with BRCA in the three subtypes. The Kaplan-Meier analysis illustrated that survival prognosis was significantly different among the three subtypes, and subtype 1 had the best prognosis, however, subtype 3 had the worst prognosis (**Figure 3B**). The expression level of PRGs and the clinical information are presented in a heatmap to display their distribution difference (**Figure 3C**). The distribution number and comparison of clinical characteristics in different subtypes are shown in **Table 1**.

**Table 1:** Distribution of clinical information among different subtypes.

Characteristics total cases	N of case 1075	Subtype			P value
		Subtype 1 (N=254)	Subtype 2 (N=416)	Subtype 3 (N=405)	
Age(years)					
≤60	596	140	257	199	1.28E-03
>60	479	114	159	206
ER status					
Positive	584	132	179	273	2.20E-16
Negative	178	79	78	21
HER2 status					
Positive	111	43	35	33	2.38E-02
Negative	635	169	210	256
PR status					
Positive	509	109	164	236	8.64E-12
Negative	249	102	92	55
Pathologic M					
M0	892	226	331	335	5.38E-01
M1	22	4	7	11
Pathologic N					
N0	504	129	181	194	2.27E-01
N1	356	79	142	135	
N2	120	27	49	44	
N3	75	14	39	22	
Pathologic T					
T1	279	60	123	96	1.86E-02
T2	622	164	227	231
T3	133	21	56	56	
T4	38	9	9	20	
Pathologic stage					
Stage I	181	41	76	64	4.54E-01
Stage II	609	154	222	233	
Stage III	244	50	104	90	
Stage IV	20	4	6	10	
Radiation therapy					
Yes	550	115	233	202	7.44E-02
No	441	111	157	173	
Recurrence					
Yes	96	20	37	39	8.09E-01
No	808	177	331	300	

### Construction and Validation of a Prognostic Signature

Univariate cox regression and K–M survival analysis were performed on the 35 significantly DEPGs acquired to find out PRGs with significant prognosis. a total of 15 significant prognostic PRGs (IRF2, TP63, IRF1, CHMP6, GZMB, NLRP6, CHMP3, IL-18, TIRAP, GPX4, GSDMD, PYCARD, CASP4, GSDMC, CHMP2A) were screened **(** **Additional file 4 Table S4** **)**. Six optimal DEPGs (GSDMC, CHMP3, IL-18, TP63, GZMB and CHMP6) with significant prognostic value were screened out through the least absolute contraction and selection operator LASSO cox analysis **(** **Additional file 5 Figure S1** **)** to construct the prognostic signature based on the following formula:


$$\begin{array}{l} \text{RS}=\left(-0.000377234\right)*{\text{Exp}}_{\text{TP}63}+\left(0.03382393\right)*{\text{Exp}}_{\text{CHMP}3}+\left(-0.01643936\right)*{\text{Exp}}_{\text{CHMP}6}+\left(-0.005408124\right)*{\text{Exp}}_{\text{GZMB}} \end{array}$$

**Figure 4:**
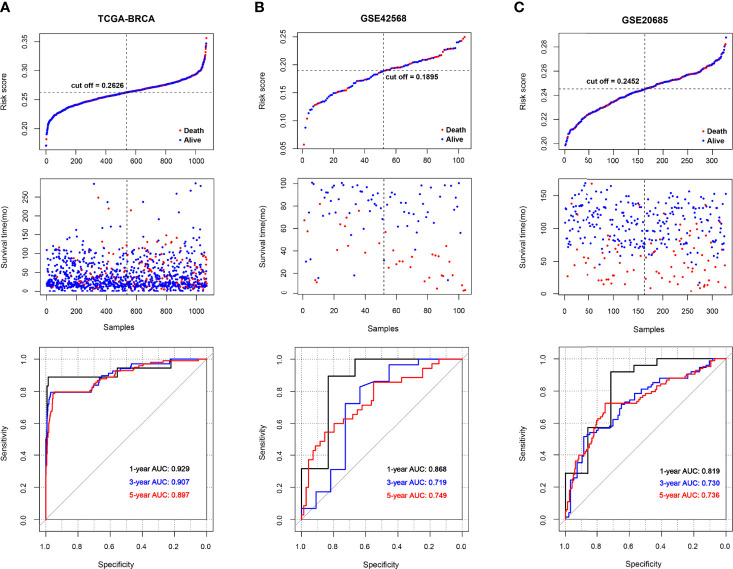
The survival status and ROC curves. **(A-C)** The survival status for each patient and the time-dependent ROC curve in TCGA **(A)**, GSE42568 **(B)**, and GSE20685 **(C)**.

**Figure 5:**
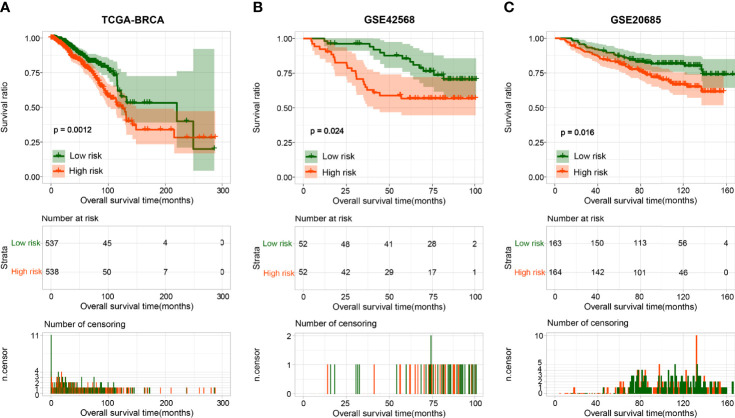
The Kaplan-Meier analysis based on the prognostic signature. The Kaplan-Meier analysis for TCGA **(A)**, GSE42568 **(B)**, and GSE20685 **(C)**. The blue and red curves represent low- or high-risk samples, respectively.

**Figure 6:**
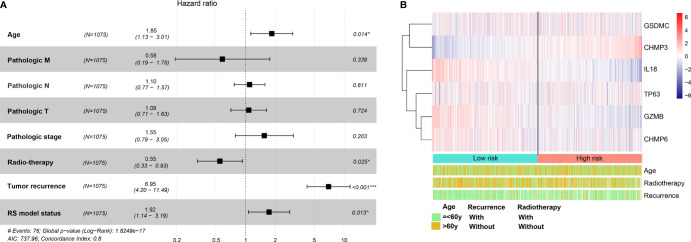
The univariate and multivariate Cox regression analysis for prognostic signature. **(A)** Forest plot for the prognosis of clinicopathologic characters. **(B)** Heatmap for the connections between the expression levels of six optimal PRGs and the distribution of the independent prognostic factors. Red: up regulated; blue: down regulated. *p < 0.05, ***p < 0.001.

BRCA patients from the TCGA-BRCA, GSE42568, and GSE20685 databases were then divided into low-risk or high-risk subgroups based on the average RS. The distribution of RS value and survival status of each risk group in the three databases is shown in **Figures 4A–C**. In addition, the time-dependent ROC curve proves that the risk assessment model is relatively stable in predicting the 1-year, 3-year, and 5-year survival rates of BRCA patients (survival AUC exceeds 0.7, **Figures 4A–C**). The Kaplan-Meier survival curve shows that the overall survival (OS) rate of the high-risk group in the three databases is significantly lower than that of the low-risk group, indicating the accuracy of the risk model in predicting survival status (**Figures 5A–C** **)**. The univariate and multivariate cox regression analysis of clinical characteristics and RS model showed that age, radiotherapy, recurrence and RS model are independent prognostic factors for BRCA patients **(** **Figure 6A** and **Table 2** **)**. The expression levels of six optimal PRGs and the distribution of the independent prognostic factors were shown in **Figure 6B**.

**Table 2:** The univariate and multivariate Cox regression analysis.

Clinical characteristics	Uni-variables cox			Multi-variables cox		
	HR	95%CI	P	HR	95%CI	P
Age (=<60/>60y)	1.964	1.423-2.710	2.88E-05	1.846	1.131-3.013	1.42E-02
Pathologic_M (M0/M1/-)	1.731	1.236-2.422	4.23E-11	0.582	0.192-1.764	3.39E-01
Pathologic_N (N0/N1/N2/N3)	1.611	1.356-1.912	2.91E-08	1.098	0.766-1.574	6.11E-01
Pathologic_T (T1/T2/T3/T4)	1.461	1.199-1.780	1.63E-04	1.078	0.711-1.634	7.24E-01
Pathologic_stage (I/II/III/IV)	2.204	1.761-2.759	3.67E-12	1.551	0.789-3.046	2.03E-01
ER status (Positive/Negative)	0.991	0.641-1.532	9.69E-01	–	–	–
HER2 status (Positive/Negative)	1.031	0.585-1.811	9.20E-01	–	–	–
PR status (Positive/Negative)	0.922	0.627-1.356	6.80E-01	–	–	–
Radio-therapy (Yes/No)	0.585	0.402-0.852	4.62E-03	0.549	0.325-0.929	2.54E-02
Recurrence (Yes/No)	7.679	5.069-11.63	2.00E-16	6.951	4.202-11.49	4.15E-14
RS model status (High/Low)	1.731	1.236-2.422	1.08E-03	1.912	1.142-3.191	1.30E-02

### Functional Pathways

**Figure 7:**
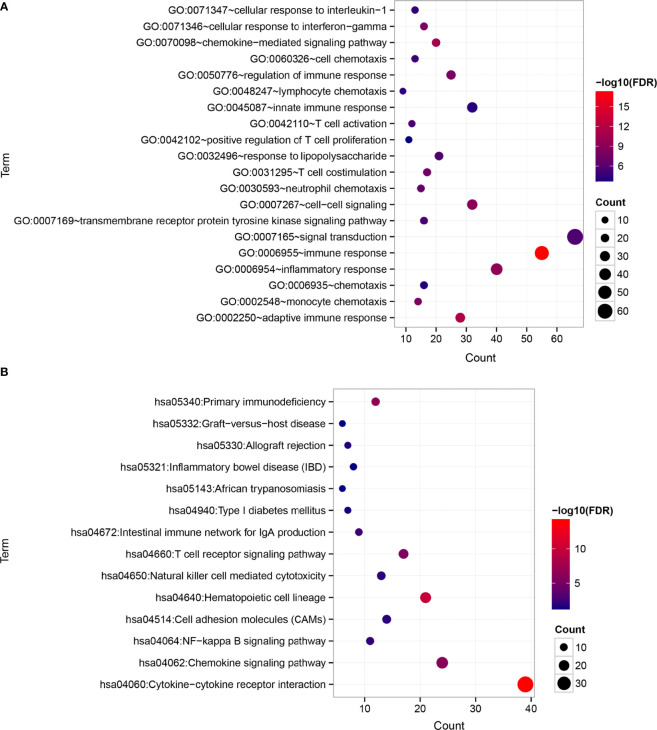
Differences in functional pathways between the risk groups. **(A)** Biological processes (BP). **(B)** KEGG signaling pathways. The horizontal axis represents the number of significantly differentially expressed genes, the vertical axis represents the item name, the size of the dots represents the number of DEGs, the color of the dots represents the enrichment significance, and the closer the color is to red, the higher the significance.

A total of 525 DEGs between the two risk groups were screened out by the limma of R (**Additional file 6 Table S5**). After that, functional enrichment analysis were performed by DAVID. 41 significantly correlated biological processes and 14 KEGG signaling pathways were enriched and top 20 of them were selected for display (**Figures 7A, B**). The results suggested that those DEGs were obviously related to several biological processes in immunity and tumor development.

### Immunity Analysis

**Figure 8:**
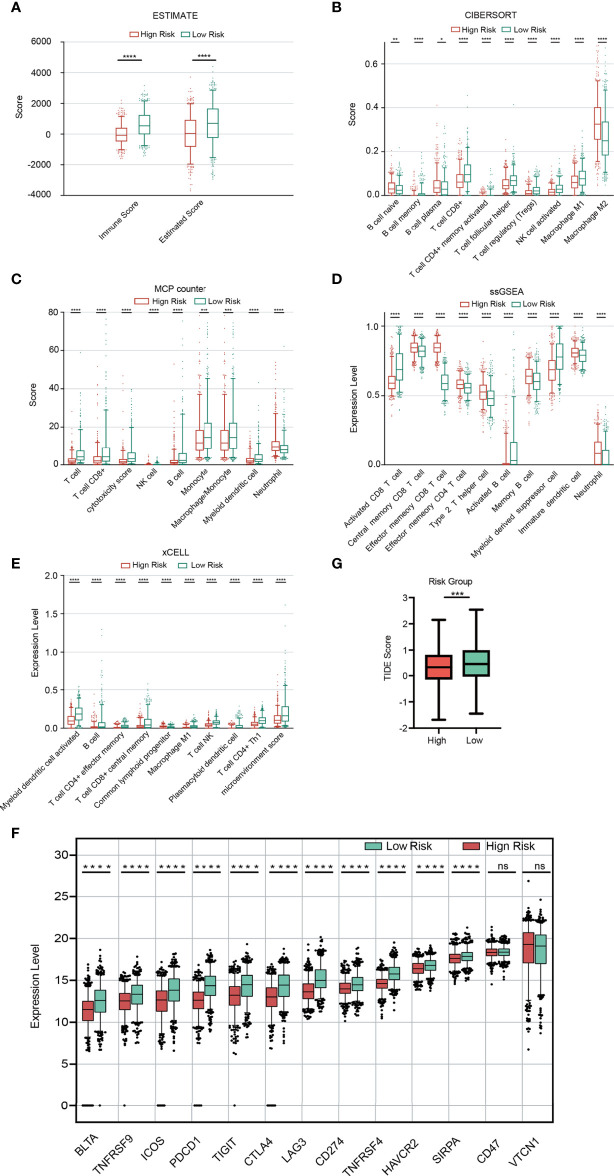
Immune analysis. **(A)** Comparison of the immune score and ESTIMATE score between the two risk groups. **(B-E)** Comparison of immune cells between the two risk groups based on Cibersort **(B)**, MCPcount **(C)**, ssGSEA **(D)** and xCELL **(E)**. Expression distribution of 13 immune checkpoint genes between the two risk groups **(F)**. TIDE score between the two risk groups **(G)**.

According to five algorithms (ssGSEA, Estimate, MCPcounter, xCELL and Cibersort), immune score, estimate score, the stromal score and the relative infiltration abundance of stromal cells and immune cells are estimated for each sample (**Additional file 7 Table S6**). The results of our analysis showed that immune score and Estimate score were higher in the low-risk group (**Figure 8A**). Based on the remaining 4 algorithms, we found that a total of 79 microenvironmental cells appeared, with significant differences. The first 10 are selected for display (**Figures 8B–E**). In view of the importance of immune checkpoints in cancer treatment, the expression of 13 checkpoint genes (BTLA, TNFRSF9, ICOS, PDCD1, TIGIT, CTLA4, LAG3, CD274, TNFRSF4, HAVCR2, SIRPA, CD47 and VTCN1) were compared. The box plot of the expression distribution of 13 immune checkpoint genes between the two risk groups is shown in **Figure 8F**. The results showed that, except for CD47 and VTCN1, there were significant differences in the other 11 genes (P<0.05). The expression of 11 genes in the high-risk group was low. The TIDE score is closely related to the response to ICB. In **Figure 8G**, the TIDE score of BRCA patients in the high-risk group is lower than that in the low-risk group, indicating that BRCA patients with higher RS are more sensitive to ICB treatment.

### Sensitivity of Chemotherapy Drugs

**Figure 9:**
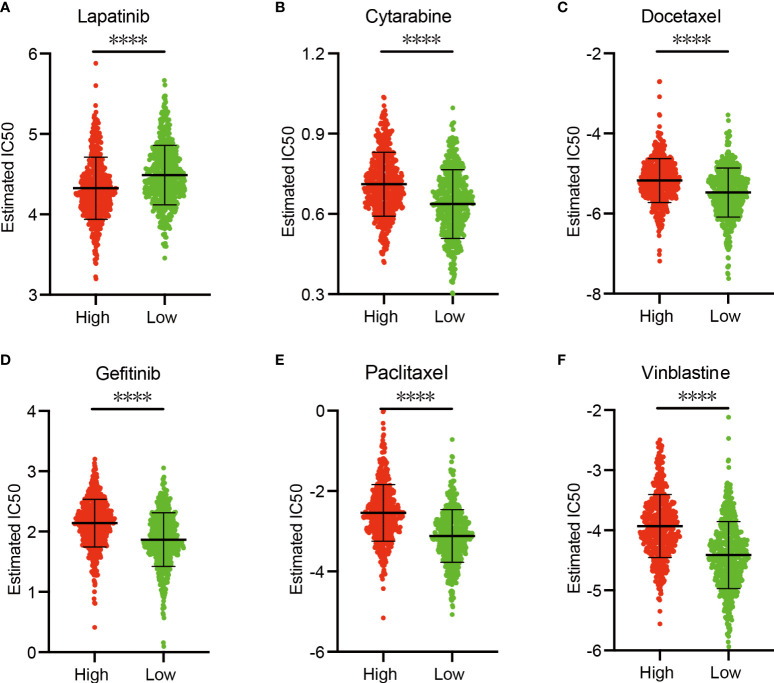
Sensitivity of chemotherapy drugs. **(A–F)** Difference in the estimated IC50 levels of Lapatinib **(A)**, Cytarabine **(B)**, Docetaxel **(C)**, Gefitinib **(D)**, Paclitaxel **(E)**, and Vinblastine **(F)**. Data are shown as means ± S.D. ns: not significant, *p < 0.05, **p < 0.01, ***p < 0.001, ****p < 0.0001.

In view of the significance of chemotherapy in BRCA treatment, we quantified the response ability of BRCA patients with different risk scores to 21 chemotherapy drugs. **Figure 9** showed the results of six commonly used BRCA chemotherapy drugs. Our data showed that the IC50 level of Lapatinib was significantly higher in the low-risk group than in the high-risk group (**Figure 9A**). In contrast, the IC50 levels of Cytarabine (**Figure 9B**), Docetaxel (**Figure 9C**), Gefitinib (**Figure 9D**), Paclitaxel (**Figure 9E**), and Vinblastine (**Figure 9F**) in the low-risk group were significantly lower than the high-risk group, indicating that BRCA patients in the low-risk group are more sensitive to these drugs.

### Expression of the Signature PRGs

**Figure 10:**
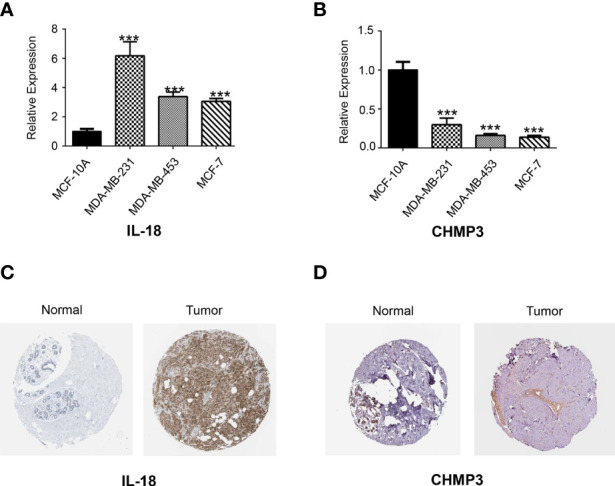
Real-time qPCR and HPA analysis. The expression of two candidate PRGs including IL-18 **(A)** and CHMP3 **(B)** in both normal breast cell line (MCF-10A) and breast cancer cell lines (MDA-MB-231, MDA-MB-453 and MCF-7) were checked by qPCR analysis. IHC staining for IL-18 **(C)** and CHMP3 **(D)** from HPA database.

To explore the expression level of the signature PRGs, we performed real-time qPCR analysis on three PRGs in different cell lines. Results suggested that CHMP3 were down-regulated in the BRCA cell lines including MDA-MB-231, MDA-MB-453 and MCF-7 compared with in the normal breast MCF-10A cell line. While IL-18 were up-regulated (**Figures 10A, B**). We also checked the IHC staining data from both normal and BRCA tissues in the HPA database. As shown in **Figures 10C, D**, expression of IL-18 was higher in BRCA tissues than normal ones, while expression of CHMP3 was lower in BRCA tissues.

## Discussion

Pyrolysis is a kind of programmed cell death accompanied by the inflammatory response, mainly triggered by activating inflammatory cysteine protease caspase-1/4/5/11 to cleave GSDMD or apoptotic cysteine protease Caspase-3 to cleave GSDME (11). Pyroptosis is involved in the occurrence and development of various diseases (38, 39). In recent years, the significance of pyroptosis in cancer has received extensive attention, accumulated many new achievements, and formed some new insights (40, 41). Chemotherapeutic agents activate Caspases to induce tumor cell death in either apoptosis or pyroptosis, depending on the expression level of GSDME in the cells. In lung cancer cells, loss of GSDME expression promotes resistance to chemotherapy, while overexpression of GSDME enhances the sensitivity of cells to chemotherapy drugs (42, 43). In HPV-infected cervical cancer cells, AIM2 plays a cancer-suppressive role by promoting pyroptosis (44). Studies have shown that the combination of low-dose PLK1 inhibitor BI2536 can enhance the sensitivity of esophageal squamous cancer cells to cisplatin. The mechanism lies in that BI2536 can inhibit the DNA damage repair pathway and promote cell pyroptosis mediated by the Caspase-3-GSDME pathway in coordination with cisplatin (45).

In order to verify the importance of pyrolysis in BRCA progression, we developed the prognostic and diagnostic models related to pyrolysis. The gene expression levels of 49 PRGs were studied in BRCA and normal tissues, and 35 PRGs were found to be differentially expressed. Then we studied the importance of these PRGs related to survival. Several PRGs that are highly expressed in BRCA tissues also predicted a poor prognosis, such as GSDMC. GSDMC has also been proved to be over-expressed in lung adenocarcinoma and function as a predictive factor for poor prognosis (46), which inspired us to put much more effort into exploring the function of GSDMC as a prognostic biomarker in BRCA. To further prove the PRGs expression signature has significant prognostic value in BRCA, we constructed a prognostic risk model through univariate and multivariate Cox regression analysis. Our research generated a signature characteristic of 6 PRGs, which could predict prognosis in BRCA patients. Among them, GSDMC firstly caught our attention. As we introduced above, Caspase-1/4/5/11 cleavage of GSDMD or cleavage of GSDME by caspase-3 has defined the canonical pyroptosis pathways. However, few studies have been focused on the GSDMC. GSDMC, also known as gasdermin C, was one member of the six human gasdermin family, of which five were reported to relate with significant biological functions, while the function of GSDMC has not been identified clearly (47). GSDMC is expressed mainly in the trachea and spleen (48). In recent reports, it also detected in the gastric epithelium (49). The full length of GSDMC before cleavage is inactive. The released N-terminal moiety binds to membranes and forms pores upon cleavage, triggering cell death (50). The most recent study of GSDMC proposed that under the condition of hypoxia and TNF-α treatment, GSDMC gene transcription was enhanced by PD-L1 and cleaved by activated caspase-8. Generated GSDMC N-terminal domain induced tumor necrosis by switch apoptosis to pyroptosis (51). This GSDMC/caspase-8-mediated cell death provides new and valuable insights into the pathway of cancer cell pyrolysis. Interestingly, in our research, caspase-8 (CASP8) also appears to be up-regulated in BRCA organizations (**Figure 2A**). A wonder whether GSDMC/caspase-8-mediated pyrolysis can be a potential therapeutic target for BRCA. It is also valuable to study whether GSDMC/caspase-8-mediated pyrolysis has a role in other types of cancer. GZMB, also named Granzyme B, take participate in the classical pyroptosis pathway. It was reported that GZMB from killer cells could cleave the GSDME directly and promote the occurrence of pyroptosis, which could further activate the anti-tumor immune response and inhibit tumor growth (52). However, the function of GZMB-related pyroptosis in BRCA remains unclear. Our results suggested that GZMB was highly expressed in BRCA tissues. In addition, the highly GZMB expression was connected with a good survival outcome, which is consistent with the conclusion proved in other cancers. Our study indicated that GZMB should also function as a promising target for BRCA prognosis. IL-18 is a chemokine that attracts basophils, neutrophils and T cells. It is released from several cell types in response to inflammatory stimuli (53, 54). When caspase-1 mediated cell pyroptosis occurred, the activated caspase-1 would cleave the precursor of IL-18 and IL-1β and release these two factors into cells. The release of IL-18 is regarded as the key signal of cell pyroptosis. We found that the IL-18 expression was up-regulated in BRCA tissues and predicted a good survival rate in BRCA. This result differs from its expression related to prognosis in other cancers. We speculate that it might be due to the inflammatory microenvironment induced by IL-18 and other inflammatory factors. Inflammasome plays a “double-edged sword” role in tumor progress. On the one hand, inflammasome, such as IL-18, could induce pyroptosis and inhibit tumor cell proliferation; On the other hand, the cumulative effect of inflammatory bodies could also build a suitable microenvironment for tumor growth. Thus, the precise condition that IL-18 participates in pyropyosis and its related prognosis in BRCA is worth discussing further issues. The relationship between CHMP6,CHMP3 and TP63 with cell pyrophosis has rarely been mentioned. Previous studies reported that CHMP6 was significantly down-regulated across several kinds of cancers including BRCA, especially in triple-negative breast cancer. Increasing understanding of these PRGs will provide a new insight into the therapeutic strategies for BRCA.

We further explored the differences in responding to ICB therapy, chemotherapy drug sensitivity between the two risk groups. Our data showed that BRCA patients with higher RS are more sensitive to ICB treatment and lapatinib, while patients in the low-risk group are more sensitive to Cytarabine, Docetaxel, Gefitinib, Paclitaxel, and Vinblastine. Our findings reveal the potential biomarkers and therapeutic targets of PRG-based risk models.

Our research still has limitations. For example, we mainly explore the functions of these PRGs through bioinformatics analysis. Therefore, further experimental data is needed to support these conclusions. Despite these limitations, our study used two validation sets to prove the effectiveness of the risk model for predicting prognosis.

## Conclusions

In conclusion, our risk model based on six PRGs identified and validated is an independent prognostic factor for BRCA patients. Through comprehensive analysis, our research results reveal the potential biomarkers and treatment targets of PRG-based risk models.

## Data Availability Statement

The original contributions presented in the study are included in the article/**Supplementary Material**. Further inquiries can be directed to the corresponding author.

## Author Contributions

NL conceived, designed, and supervised the study. YZ drafted the manuscript. JZ performed the data analysis. MB arranged the figures. YG performed the HPA analysis and *in vitro* assay. All authors contributed to the article and approved the submitted version.

## Funding

This work was supported by the National Natural Science Foundation of China (Grant No. 31900922), the Beijing Zhongwei Joint Funds of the Zhejiang Provincial Natural Science Foundation of China (Grant No. LBY22H200007) and the National Natural Science Foundation of China (Grant No. 82003838).

## Conflict of Interest

The authors declare that the research was conducted in the absence of any commercial or financial relationships that could be construed as a potential conflict of interest.

## Publisher’s Note

All claims expressed in this article are solely those of the authors and do not necessarily represent those of their affiliated organizations, or those of the publisher, the editors and the reviewers. Any product that may be evaluated in this article, or claim that may be made by its manufacturer, is not guaranteed or endorsed by the publisher.

## Supplementary Material

Additional file 1 Table S1 The primer sequences in PCR analysis.

Additional file 2 Table S2 Protein–protein interactions.

Additional file 3 Table S3 Co-expression network.

Additional file 4 Table S4 15 significant prognostic PRGs.

Additional file 5 Figure S1 λ selection diagram.

Additional file 6 Table S5 DEGs of the two risk groups.

Additional file 7 Table S6 Immunity analysis.

## Abbreviations

BRCA, Breast cancer; TCGA, The Cancer Genome Atlas; PRG, Pryroptosis-relate gene; LASSO, Least absolute shrinkage and selection operator; Quantitative PCR, qPCR; DEG, Differentially expressed gene; TIDE, Tumor immune dysfunction and exclusion; PCC, Pearson correlation coefficient; DEPG, Differentially expressed PRG; RS, risk score; ssGSEA, Single-sample gene set enrichment analysis; ICB, Immune checkpoint blockade; IHC, immunohistochemical; RS, Risk score; PPI, protein-protein interaction; OS, Overall survival.
