# Supplementary figures and images for: Effect of Pyroptosis-Related Genes on the Prognosis of Breast Cancer
Source: Front Oncol. 2022 Jul 25;12:948169. doi: 10.3389/fonc.2022.948169 (PMC9357945; doi:10.3389/fonc.2022.948169)

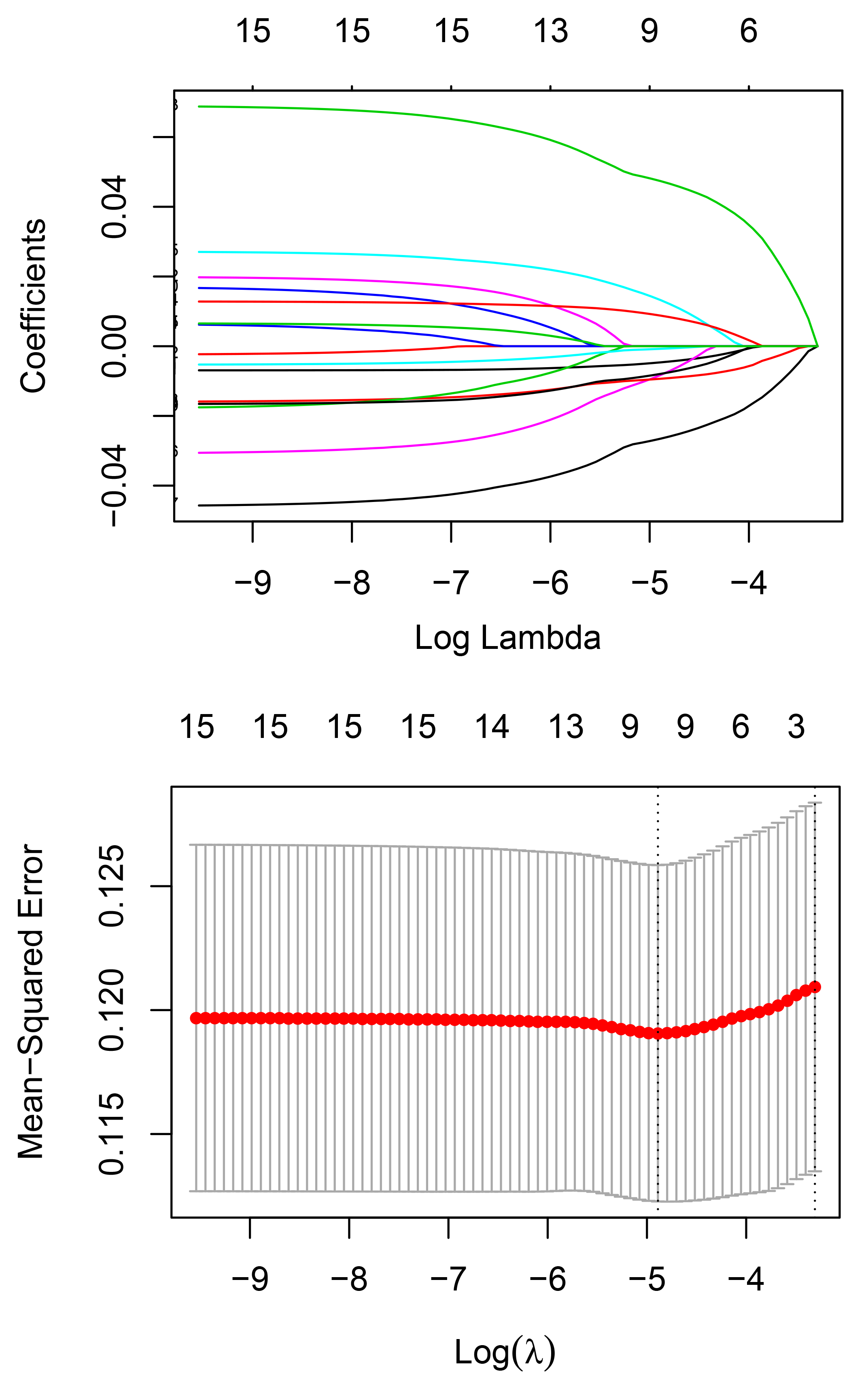

Supplement: Additional file 5 Figure S1 — λ selection diagram. [file Image_1.tif]
